# Antidepressant use during pregnancy and puberty timing in the offspring: results from the Norwegian Mother, Father, and Child Cohort Study

**DOI:** 10.1093/ije/dyag240

**Published:** 2026-11-04

**Authors:** Alessandro Riva, Olav Spigset, Hedvig Nordeng, Per Magnus, Nhung T H Trinh

**Affiliations:** Pharmacoepidemiology and Drug Safety Research Group, Department of Pharmacy, Faculty of Mathematics and Natural Sciences, University of Oslo, Oslo, Norway; University of Milano, Milan, Italy; Department of Clinical Pharmacology, St. Olav University Hospital, Trondheim, Norway; Department of Clinical and Molecular Medicine, Faculty of Medicine and Health Sciences, Norwegian University of Science and Technology, Trondheim, Norway; Pharmacoepidemiology and Drug Safety Research Group, Department of Pharmacy, Faculty of Mathematics and Natural Sciences, University of Oslo, Oslo, Norway; Centre for Fertility and Health, Norwegian Institute of Public Health, Oslo, Norway; Pharmacoepidemiology and Drug Safety Research Group, Department of Pharmacy, Faculty of Mathematics and Natural Sciences, University of Oslo, Oslo, Norway

**Keywords:** prenatal antidepressant, puberty, depression, anxiety

## Abstract

**Background:**

Preclinical studies suggest perinatal antidepressant exposure may alter pubertal timing, but human evidence is lacking.

**Objective:**

To investigate whether maternal antidepressant exposure is associated with pubertal timing in human offspring.

**Study design:**

We used data from the Norwegian Mother, Father, and Child Cohort Study linked with the Medical Birth Registry of Norway, including 1396 singleton births to women with depression/anxiety before pregnancy. Group-based trajectory modeling was used to identify four antidepressant exposure trajectories: continuers (*n* = 49, 3.5%), early discontinuers (*n* = 140, 10.0%), late discontinuers (*n* = 51, 3.7%), and non-exposed (*n* = 1156, 82.8%). The Pubertal Development Scale with five items assessed at 14 years allowed us to classify the outcome variable as early, normal, and delayed puberty. Modified Poisson regression with generalized overlap weighting was used to estimate weighted risk ratios (wRR) and their 95% confidence interval (CI).

**Results:**

In total, delayed puberty occurred in 17 children (1.2%), mainly in males. Early puberty occurred in 631 (45.2%), more frequently among females. Compared with unexposed children, children born to continuers, early discontinuers, and late discontinuers had lower point estimates of risk of early puberty (wRR range 0.77–0.82). When early discontinuers were the comparator, estimates of early puberty risk were attenuated closer to the null (wRR range 1.00–1.07), with differences observed between males and females. Crude estimates, however, suggested higher risk of delayed puberty.

**Conclusion:**

Children born to mothers who continued antidepressants or discontinued late in pregnancy did not have increased risk of early puberty, but the risk of delayed puberty might be higher.

Key Messages

- Preclinical studies suggest perinatal antidepressant exposure may alter pubertal timing.
- Delayed puberty (i.e. pre-pubertal at age 14 years) was rare while early puberty (i.e. post-pubertal at age 14 years) was common among offspring of women with pre-pregnancy depression/anxiety.
- Offspring born to mothers who continued antidepressants or discontinued late in pregnancy did not have increased risk of early puberty.

## Introduction

Depression and anxiety are the most common mental disorders that pose significant burden among women of childbearing age. In moderate to severe cases, antidepressants are given as first-line pharmacological treatment [1]. The prevalence of antidepressant use during pregnancy is estimated to range between 2% and 13% [2]. Because antidepressants cross both the placenta and the blood–brain barrier, concerns have been raised about potential disruption of fetal developmental processes and subsequent short- and long-term consequences. Although short-term and neurodevelopmental outcomes have been widely studied, evidence on long-term pubertal development remains limited.

Puberty is the key development stage of transition from childhood to adult life, with the achievement of adult height and body proportions, the development of external sexual characteristics and the capacity to reproduce. Abnormal timing (very early or very late) of pubertal development has been linked to increased risks of obesity, type 2 diabetes, cardiovascular disease, hormone-related cancers, and psychosocial difficulties including depression, anxiety, low-esteem, and behavioral problems [3]. The mechanisms underlying abnormalities in pubertal timing are multifactorial and reflect complex interactions between genetic factors, dysregulation of the hypothalamic–pituitary–gonadal axis, metabolic factors (e.g. adiposity), environmental exposures, and psychosocial influences, many of which remain incompletely understood [4]. Several preclinical studies have examined the effects of maternal exposure to antidepressants during gestation and lactation on pubertal development and reproductive health in offspring. In male rats, exposure to antidepressants during pregnancy and lactation disrupts spermatogenesis, testicular architecture, and hormonal homeostasis [5]. In female rats, perinatal antidepressant exposure may be associated with altered estrous cyclicity, delayed or precocious puberty, disrupted ovarian gene expression, and impaired follicular maturation [6]. Given these preclinical findings, it is crucial to investigate whether similar associations are observed in human populations.

The aim of this study was to investigate whether prenatal antidepressant exposure was associated with pubertal stage of the offspring at 14 years of age using data from the Norwegian Mother, Father, and Child Cohort Study (MoBa).

## Methods

### Data sources

The study used data obtained from MoBa, linked with records in the Medical Birth Registry of Norway (MBRN). Linkage of data was carried out using the unique personal identifier number of each citizen of Norway.

MoBa is a nationwide, prospective population-based pregnancy study conducted by the Norwegian Institute of Public Health [7, 8]. Pregnant women were recruited from all over Norway in 1999–2008 through a postal invitation in connection with a publicly offered routine ultrasound at 17–18 weeks of gestation. The MoBa cohort includes approximately 114 500 children, 95 200 mothers, and 77 300 fathers. In MoBa, data were collected prospectively via two prenatal questionnaires, at week 17 (Q1) and week 30 (Q3), as well as multiple postnatal self-administered questionnaires at different child ages up to adolescence. Fathers-to-be also completed a prenatal questionnaire in week 17 (Q-Father). All MoBa questionnaires are available online [9]. The current study is based on version 12 of the quality-assured MoBa data files released for research purposes.

The MBRN is a national health registry that collects mandatory standardized notification forms of all live births, stillbirths and induced abortions after week 12 in Norway since 1967 [10].

### Study population

We included singleton pregnancies with liveborn children after gestational week 32 (i.e. around the completion of Q3) in women who self-reported depressive and/or anxiety disorders before pregnancy in Q1, complemented with diagnostic records from MBRN (ICD-10 codes: F32, F33, F40, F41). Details about depression/anxiety reporting are presented in Supplementary Appendix 1. Children born to multiple pregnancies were excluded since they experience different intrauterine conditions, growth patterns, and hormonal influences which may influence pubertal development. We required that Q1, Q3, and Q14years had been returned so that information regarding perinatal medication use and puberty outcome were available. We excluded pregnancies with missing data on all questions related to puberty outcomes.

### Definition of exposure

Information on the type and timing of antidepressant use was obtained from Q1 and Q3. Women were asked to report medication use for numerous chronic, short-term, and pregnancy-related conditions in an open textbox. In Q1, medication use was documented for 6 months before pregnancy and during pregnancy in 4-week intervals: from week zero until after week 13 (0–4, 5–8, 9–12) and from week 13 until the completion of the first questionnaire. In Q3, medication use was documented during pregnancy in 4-week intervals: from week 13 until after week 29 (13–16, 17–20, 21–24, 25–28) and from 29 until the completion of Q3.

To characterize longitudinal patterns of maternal antidepressant use, we employed group-based trajectory modeling (GBTM) [11]. GBTM is a semi-parametric, finite mixture modeling approach that identifies latent subpopulations with distinct developmental trajectories, enabling the detection of heterogeneous patterns of exposure across time. Unlike binary exposure indicators or cross-sectional summaries, GBTM captures the dynamic and evolving nature of antidepressant use, thereby enhancing analytical precision and clinical interpretability [12]. We performed GBTM across nine time points: pre-pregnancy, weeks 0–4, 5–8, 9–12, 13–16, 17–20, 21–24, 25–28, 29+.

We fitted models specifying between two and six clusters and evaluated model fit using the Bayesian Information Criterion (BIC), selecting the model with the lowest BIC as an indicator of optimal balance between model fit and parsimony. We additionally considered entropy values to assess classification accuracy, preferring models with entropy greater than 0.80 to ensure adequate separation between trajectory groups. Model selection also accounted for the interpretability, clinical plausibility, and sufficient size of each trajectory group.

### Puberty timing

In the 14-year questionnaire, children reported their pubertal status using the Self-rating Scale for Pubertal Development Scale (PDS) [13]. The PDS is an adaptation of an interview-based puberty-rating scale originally developed by Petersen *et al*. and serves as a useful tool for assessing pubertal development in large-scale studies requiring non-invasive measures. The scale includes five items assessing general markers of pubertal development, including growth spurt (length), body hair growth, and skin changes (pimples), as well as sex-specific characteristics such as voice deepening and facial hair for boys, and breast development and menstruation for girls. The PDS has demonstrated acceptable validity compared with clinician-rated Tanner staging and good internal consistency and test–retest reliability in adolescent populations [14, 15]. To approximate Tanner stages, pubertal development was classified using PDS scores into pre-pubertal, early pubertal, mid-pubertal, late pubertal, and post-pubertal (details in Supplementary Appendix 2) [15]. Although norms for pubertal onset vary between ethnic groups, the accepted age limits for Tanner genital stage 2 (G2) in boys are 9–14 years, and for Tanner breast stage 2 (B2) in girls are 8–13 years [16]. Accordingly, individuals classified as pre-pubertal at age 14 years were identified as having delayed puberty while those classified as post-pubertal at age 14 years were identified as having early puberty.

### Confounders

A sufficient set of variables was examined and selected using a Directed Acyclic Graphs (DAGs) generated with DAGitty (Supplementary Fig. 1), which guided adjustments in the analysis. Maternal confounders included maternal age at delivery, maternal civil status, maternal income, maternal education level, parity, history of abortions, and miscarriages, BMI at conception, smoking before pregnancy, nicotine use before pregnancy, alcohol consumption before pregnancy, illicit substance before pregnancy, folate before pregnancy, self-reported depression before pregnancy, self-reported anxiety before pregnancy, lifetime history (LTH) of major depression, co-medication with opioids, benzodiazepine derivates, hypnotics and sedatives, antipsychotics and antiepileptics before pregnancy, obstetric comorbidity index (details in Supplementary Appendix 3). Paternal confounders include paternal age, paternal income, paternal education level, and LTH of major depression. Definitions of these variables were presented in Supplementary Appendix 4.

### Handling of missing data

Missing data were handled using multiple imputation by chained equations (MICE) under the missing at random assumption [17]. The imputation model was implemented in Stata using the mi impute chained command. The imputation model included all variables used in the main analysis related to the exposure, outcome, and potential confounders, as well as auxiliary variables predictive of missingness. In our dataset, approximately 50% of observations had at least one missing value among the confounders. Fifty multiple imputed datasets (*m* = 50) were generated with an iterative phase of 10 cycles to ensure stability, precision, valid statistical inference, and convergence of the imputation process.

### Confounding adjustment

To account for confounding due to the non-random nature of exposure to antidepressants during pregnancy, we applied Generalized Overlap Weighting (GOW), a propensity score-based approach designed to create a pseudo-population in which the distribution of observed covariates is independent of treatment assignment, thereby mimicking the conditions of a randomized controlled trial [18]. Compared with other propensity score approaches such as inverse probability of treatment weighting or matching, GOW emphasizes individuals with the greatest clinical equipoise (i.e. overlapping propensity scores) thus reduces the influence of extreme weights, and improves covariate balance while retaining the full analytic sample [18]. The generalized propensity score, defined as the conditional probability of treatment assignment given a set of observed covariates, was estimated using multinomial logistic regression including all relevant confounders. Covariate balance post-weighting was assessed using standardized mean differences (SMDs), with values below 0.1 considered indicative of sufficient balance. To account for residual imbalance, covariates with an SMD above the threshold were additionally included in the outcome model.

### Statistical analysis

We used modified Poisson regression models with robust standard errors to estimate Risk Ratios (RR) for binary outcome variables (early/delayed puberty vs normal puberty). Models incorporated overlap weights via the pweight option. All analyses were performed in overall and then stratified by child sex.

To improve the power in some comparisons and facilitate the comparison with studies adopting binary classification of exposure, we grouped continuers and late discontinuers as exposed group during pregnancy and estimate the risk of outcomes compared to unexposed and early discontinuers.

Data management and analyses were performed using STATA 18 statistical software (Stata Corporation, College Station, TX).

## Results

We included 1396 children (750 females, 52.3% and 666 males, 47.7%) in this study (Fig. 1). Among them, 17 (1.2%) were classified as having delayed puberty while 631 (45.2%) were classified as having early puberty. Among children with early puberty, two-thirds were females (*n* = 434, 68.8%) while 16/17 children classified as having delayed puberty were male. The GBTM analyses identified four antidepressant treatment trajectories (Fig. 2 and Supplementary Appendix 5): *continuers* (*n* = 49, 3.5%—those reported using antidepressant before and throughout pregnancy), *early discontinuers* (*n* = 140, 10.0%—those reported using antidepressant before pregnancy but discontinued around pregnancy start), *late discontinuers* (*n* = 51, 3.7%—those reported using antidepressant before pregnancy and discontinued toward the end of the pregnancy), and *non-exposed* (*n* = 1156, 82.8%—those reported not using antidepressant both before and during pregnancy). The characteristics of the overall cohort and treatment trajectory were presented in Table 1. SSRIs were the most commonly used antidepressants both before and during pregnancy. Mothers who continued antidepressants in pregnancy were slightly older, more likely to report lifetime history of major depression and had slightly higher depressive symptom scores. The differences in the selected confounders across comparison groups were generally well-balanced after weighting across all imputations.

**Figure 1:**
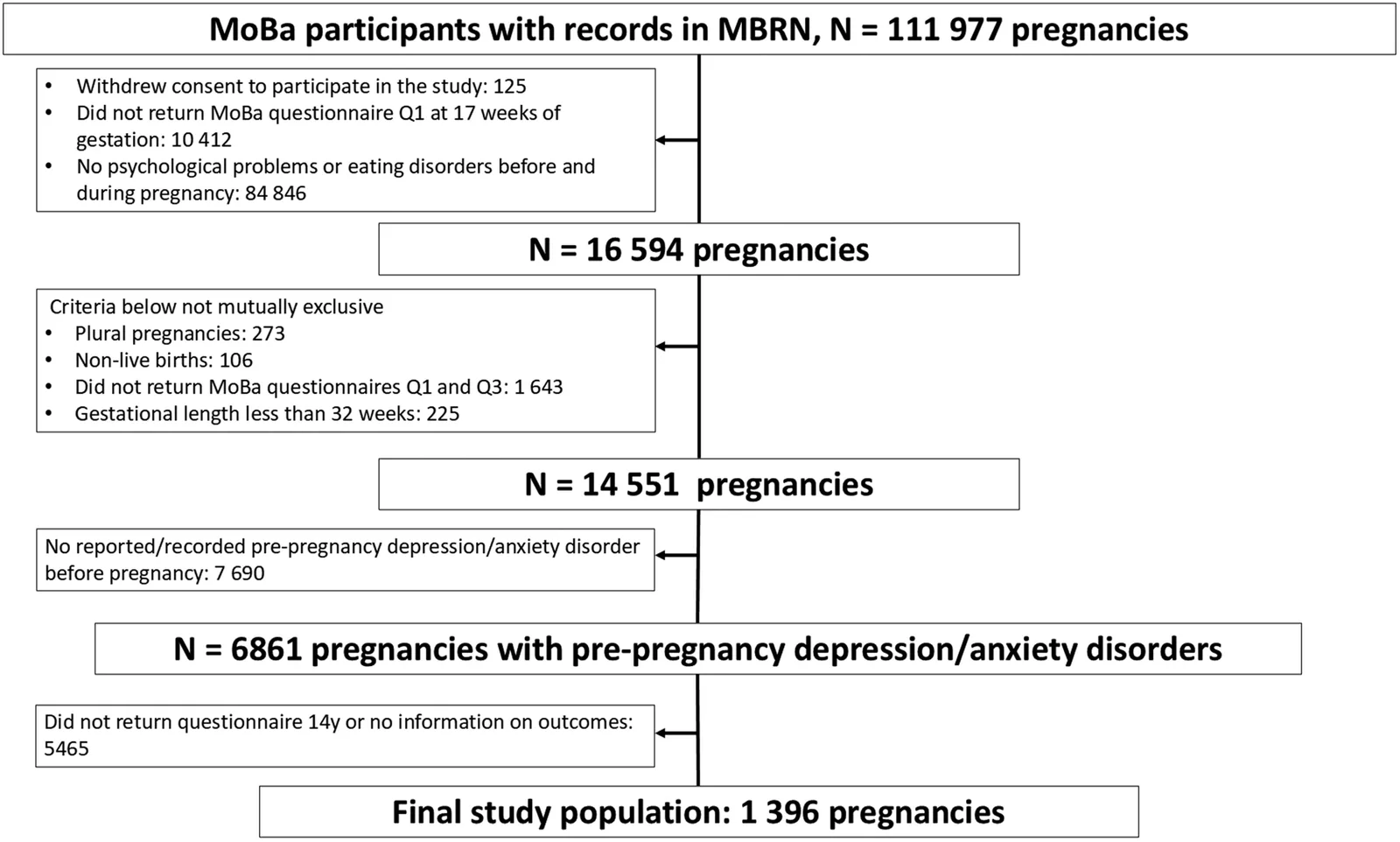
Flowchart of study population selected from the MoBa and MBRN.

**Figure 2:**
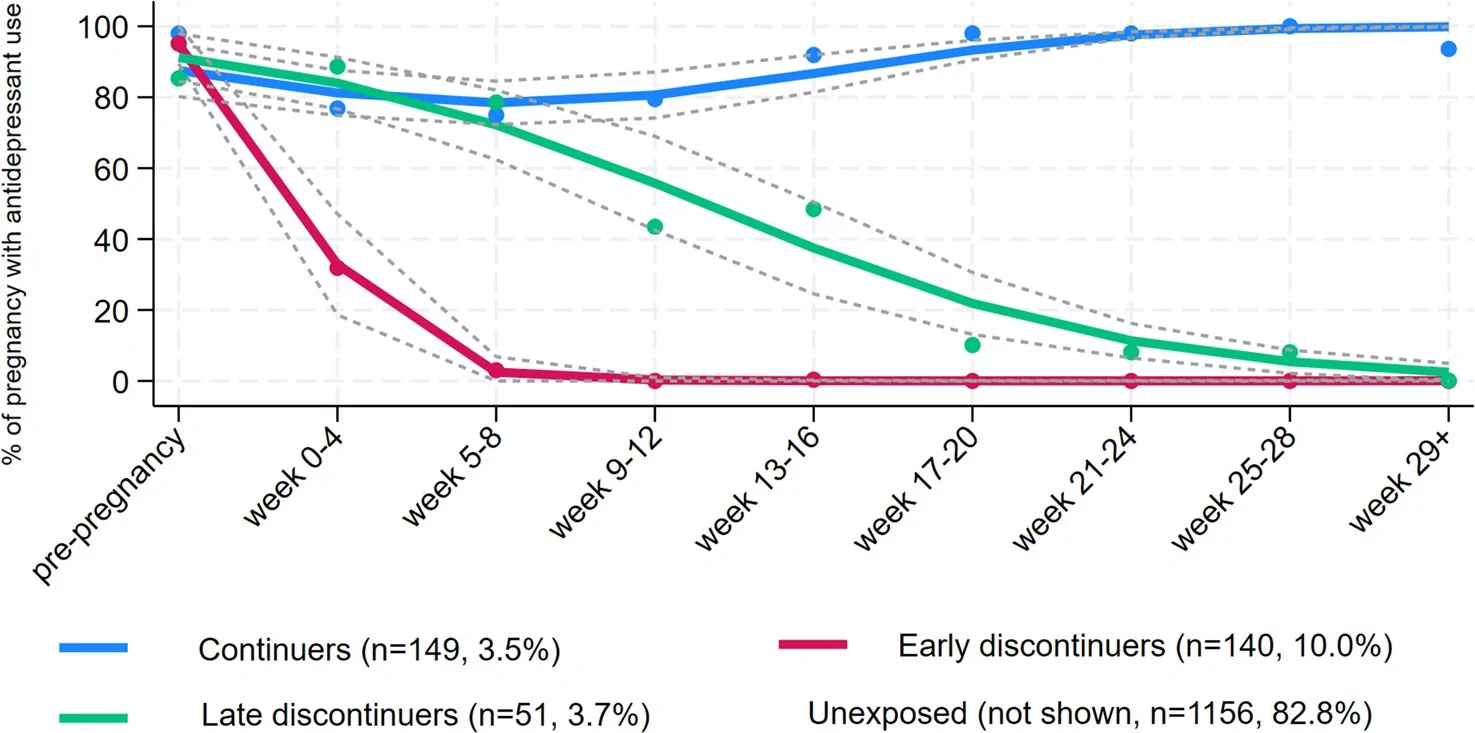
Antidepressant treatment trajectories between 6 months prior to pregnancy and 29 weeks into pregnancy.

**Table 1:** Characteristics of the study population, in overall and by antidepressant exposure trajectories.^a^

	Continuers	Early discontinuers	Late discontinuers	Unexposed	Total
**N**	49 (3.5%)	140 (10.0%)	51 (3.7%)	1156 (82.8%)	1396 (100%)
**Maternal age (years, mean [SD])**	32.0 (5.1)	29.7 (5.0)	30.5 (5.2)	30.8 (4.7)	30.7 (4.8)
**Maternal age categories**					
* <25 years*	4 (8.2%)	20 (14.3%)	7 (13.7%)	112 (9.7%)	143 (10.2%)
* 25–35 years*	33 (67.3%)	104 (74.3%)	36 (70.6%)	865 (74.8%)	1038 (74.4%)
* > =36*	12 (24.5%)	16 (11.4%)	8 (15.7%)	179 (15.5%)	215 (15.4%)
**Parity**					
* Primiparous*	26 (53.1%)	78 (55.7%)	28 (54.9%)	601 (52.0%)	733 (52.5%)
* Multiparous*	23 (46.9%)	62 (44.3%)	23 (45.1%)	555 (48.0%)	663 (47.5%)
Previous miscarriages or abortions	8 (21.1%)	29 (25.4%)	9 (20.0%)	257 (26.2%)	303 (25.7%)
Length of gestation in weeks	38.9 (1.8)	39.8 (1.2)	39.2 (1.5)	39.6 (1.6)	39.6 (1.6)
Prematurity	6 (12.2%)	<5	<5	60 (5.2%)	71 (5.1%)
**Maternal BMI at conception**					
* Underweight*	<5	8 (5.9%)	5 (10.0%)	31 (2.8%)	45 (3.3%)
* Normal weight*	27 (60.0%)	80 (59.3%)	32 (64.0%)	744 (66.2%)	883 (65.2%)
* Overweight*	11 (24.4%)	29 (21.5%)	9 (18.0%)	245 (21.8%)	294 (21.7%)
* Obese*	6 (13.3%)	18 (13.3%)	4 (8.0%)	104 (9.3%)	132 (9.7%)
**Smoking before pregnancy**	18 (38.3%)	48 (38.7%)	29 (58.0%)	340 (31.7%)	435 (33.7%)
Nicotine use before pregnancy	<5	6 (4.3%)	5 (9.8%)	95 (8.2%)	108 (7.7%)
Alcohol before pregnancy	12 (25.5%)	42 (30.9%)	14 (28.0%)	350 (31.6%)	418 (31.1%)
Illicit substance use before pregnancy	<5	<5	<5	8 (0.7%)	13 (0.9%)
Folate use before pregnancy	23 (46.9%)	61 (43.6%)	20 (39.2%)	623 (53.9%)	727 (52.1%)
Folate use during pregnancy	41 (83.7%)	118 (84.3%)	47 (92.2%)	1038 (89.8%)	1244 (89.1%)
Maternal depressive symptom checklist score in early pregnancy (mean (SD))	1.90 (0.64)	1.67 (0.57)	1.75 (0.62)	1.52 (0.55)	1.56 (0.56)
Maternal depressive symptom checklist score in late pregnancy (mean (SD))	1.76 (0.64)	1.62 (0.57)	1.65 (0.48)	1.47 (0.45)	1.50 (0.48)
Life-time history of major depression in mothers	44 (93.6%)	121 (89.0%)	45 (91.8%)	905 (82.0%)	1115 (83.5%)
**Obstetric comorbidity index**					
* 0*	29 (59.2%)	96 (68.6%)	31 (60.8%)	711 (61.5%)	867 (62.1%)
* 1–2*	14 (28.6%)	28 (20.0%)	17 (33.3%)	315 (27.2%)	374 (26.8%)
* 3–5*	6 (12.2%)	16 (11.4%)	<5	125 (10.8%)	150 (10.7%)
* >5*	0 (0.0%)	0 (0.0%)	0 (0.0%)	5 (0.4%)	5 (0.4%)
**Co-medication with psychotropics**					
* Opiods analgesics*	0 (0.0%)	6 (4.3%)	<5	19 (1.6%)	27 (1.9%)
* Benzodiazedine derivates*	5 (10.2%)	<5	<5	22 (1.9%)	34 (2.4%)
* Hypnotics and sedatives*	<5	10 (7.1%)	<5	20 (1.7%)	33 (2.4%)
* Antipsychotics*	<5	<5	<5	8 (0.7%)	12 (0.9%)
* Antiepileptics*	<5	<5	<5	5 (0.4%)	10 (0.7%)
* No*	39 (79.6%)	119 (85.0%)	40 (78.4%)	1082 (93.6%)	1280 (91.7%)
**Paternal age categories**					
* <25 years*	<5	8 (5.8%)	5 (10.2%)	46 (4.0%)	61 (4.4%)
* 25–35 years*	27 (55.1%)	87 (62.6%)	27 (55.1%)	738 (64.3%)	879 (63.5%)
* 36–46 years*	20 (40.8%)	41 (29.5%)	16 (32.7%)	331 (28.8%)	408 (29.5%)
* >46 years*	0 (0.0%)	<5	<5	33 (2.9%)	37 (2.7%)
Paternal depressive symptom checklist score (mean (SD))	1.14 (0.19)	1.17 (0.23)	1.24 (0.37)	1.18 (0.30)	1.18 (0.28)
Life-time history of major depression in father	9 (24.3%)	25 (22.9%)	10 (24.4%)	185 (19.2%)	229 (19.9%)
**Child sex**					
* Female*	27 (55.1%)	66 (47.1%)	29 (56.9%)	608 (52.6%)	730 (52.3%)
* Male*	22 (44.9%)	74 (52.9%)	22 (43.1%)	548 (47.4%)	666 (47.7%)
**Civil status**					
* Married*	23 (46.9%)	60 (43.5%)	21 (41.2%)	587 (51.3%)	691 (50.0%)
* Co-habitant*	21 (42.9%)	66 (47.8%)	25 (49.0%)	509 (44.5%)	621 (44.9%)
* Unmarried/Single*	5 (10.2%)	12 (8.7%)	5 (9.8%)	46 (4.0%)	68 (4.9%)
* Divorced/Separated/Widow*	0 (0.0%)	0 (0.0%)	0 (0.0%)	<5	<5
**Mother income**					
* Low*	16 (33.3%)	50 (37.0%)	20 (39.2%)	351 (31.0%)	437 (32.0%)
* Average*	27 (56.2%)	72 (53.3%)	24 (47.1%)	639 (56.4%)	762 (55.8%)
* High*	5 (10.4%)	9 (6.7%)	5 (9.8%)	112 (9.9%)	131 (9.6%)
* Missing*	0 (0.0%)	<5	<5	30 (2.7%)	36 (2.6%)
**Paternal income**					
* Low*	7 (15.2%)	13 (9.9%)	5 (11.1%)	126 (11.4%)	151 (11.4%)
* Average*	25 (54.3%)	82 (62.6%)	31 (68.9%)	618 (55.8%)	756 (56.8%)
* High*	14 (30.4%)	34 (26.0%)	8 (17.8%)	352 (31.8%)	408 (30.7%)
* Missing*	0 (0.0%)	<5	<5	12 (1.1%)	15 (1.1%)
**Maternal education**					
* Secondary school*	<5	<5	<5	30 (2.8%)	39 (3.0%)
* High school*	16 (34.8%)	45 (34.4%)	19 (38.0%)	320 (29.7%)	400 (30.6%)
* University*	28 (60.9%)	81 (61.8%)	29 (58.0%)	729 (67.6%)	867 (66.4%)
**Paternal education**					
* Secondary school*	<5	6 (4.5%)	<5	50 (4.5%)	59 (4.4%)
* High school*	22 (46.8%)	62 (47.0%)	27 (54.0%)	473 (42.6%)	584 (43.6%)
* University*	23 (48.9%)	64 (48.5%)	22 (44.0%)	587 (52.9%)	696 (52.0%)
**Type of antidepressants before pregnancy**					
* SSRI*	38 (77.6%)	110 (78.6%)	35 (68.6%)	–	–
* SNRI*	6 (12.2%)	13 (9.3%)	7 (13.7%)	–	–
* TCA*	<5	7 (5.0%)	<5	–	–
* Others*	<5	11 (7.9%)	<5	–	–
**Type of antidepressants during pregnancy**					
* SSRI*	40 (81.6%)	37 (26.4%)	41 (80.4%)	–	–
* SNRI*	7 (14.3%)	5 (3.6%)	8 (15.7%)	–	–
* TCA*	<5	<5	<5	–	–
* Others*	6 (12.2%)	<5	–	–	–

a SSRI, selective serotonin reuptake inhibitors; SNRI, serotonin-noradrenaline reuptake inhibitors; TCA, tricyclic antidepressants.

### Risk of early puberty

Both crude and weighted models suggested lower point estimates of early puberty among children born to *continuers, early discontinuers*, and *late discontinuers* compared to *unexposed children* (wRR = 0.82, 95% CI: 0.56–1.21, wRR = 0.77, 95% CI: 0.58–1.03 and wRR = 0.77, 95% CI: 0.50–1.17, respectively) of the estimates were close to the null when *continuers* and *late discontinuers* were compared to *early discontinuers* (wRR = 1.07, 95% CI: 0.67–1.71 and wRR = 1.00, 95% CI: 0.61–1.64, respectively)—see Table 2 for details.

**Table 2:** RR of early puberty vs normal puberty among offspring across prenatal antidepressant exposure trajectories.

Comparison	Event rates	Unweighted relative risk	Weighted relative risk
**Overall**			
* *Continuers vs unexposed	18/49 vs 538/1156	0.80 (0.55, 1.15)	0.82 (0.56, 1.21)
* *Early discontinuers vs unexposed	58/140 vs 538/1156	0.89 (0.72, 1.09)	0.77 (0.58, 1.03)
* *Late discontinuers vs unexposed	17/51 vs 538/1156	0.74 (0.50, 1.09)	0.77 (0.50, 1.17)
* *Continuers vs early discontinuers	18/49 vs 58/140	0.90 (0.59, 1.36)	1.07 (0.67, 1.71)
* *Late vs early discontinuers	17/51 vs 58/140	0.83 (0.54, 1.28)	1.00 (0.61, 1.64)
**Female**			
* *Continuers vs unexposed	14/27 vs 371/608	0.85 (0.59, 1.23)	0.94 (0.59, 1.50)
* *Early discontinuers vs unexposed	37/66 vs 371/608	0.93 (0.75, 1.16)	0.86 (0.61, 1.23)
* *Late discontinuers vs unexposed	12/29 vs 371/608	0.68 (0.44, 1.05)	0.70 (0.40, 1.22)
* *Continuers vs early discontinuers	14/27 vs 37/66	0.91 (0.60, 1.39)	1.09 (0.61, 1.93)
* *Late vs early discontinuers	12/29 vs 37/66	0.73 (0.45, 1.18)	0.81 (0.43, 1.55)
**Male**			
* *Continuers vs unexposed	<5/22 vs 167/548	0.61 (0.25, 1.49)	0.62 (0.24, 1.63)
* *Early discontinuers vs unexposed	21/74 vs 167/548	0.91 (0.62, 1.33)	0.71 (0.44, 1.14)
* *Late discontinuers vs unexposed	5/22 vs 167/548	0.80 (0.37, 1.73)	0.89 (0.40, 1.97)
* *Continuers vs early discontinuers	<5/22 vs 21/74	0.67 (0.26, 1.75)	0.88 (0.31, 2.53)
* *Late vs early discontinuers	5/22 vs 21/74	0.88 (0.38, 2.05)	1.26 (0.51, 3.11)

The stratified analyses by child sex yielded similar results in the comparison with *unexposed children*. However, compared to male children born to *early discontinuers*, male children born to *continuers* have lower point estimate of risk of early puberty (wRR = 0.88, 95% CI: 0.31–2.53) while male children born to *late discontinuers* had higher point estimate of risk (wRR = 1.26, 95% CI: 0.51–3.11). Conversely, compared to female children born to *early discontinuers,* female children born to *continuers* have slightly higher point estimate of risk of early puberty (wRR = 1.09, 95% CI: 0.61–1.93) while female children born to *late discontinuers* had lower point estimate of risk (wRR = 0.81, 95% CI: 0.43–1.55). However, the 95% CIs were highly overlapping.

### Risk of delayed puberty

Most delayed puberty cases were in children born to mothers who were not exposed to antidepressants; thus, we did not run weighted analyses for this outcome. However, we noted higher crude risks of delayed puberty in children born to *continuers* and *late discontinuers* versus *unexposed* and *early discontinuers* (Supplementary Table 1).

### Additional analysis

Children exposed to antidepressants during pregnancy (i.e. continuers and late discontinuers combined) had a lower risk of early puberty when compared to *unexposed children* (wRR = 0.80, 95% CI: 0.60–1.07). However, the risk reduction was attenuated much closer to the null when children born to *early discontinuers* were used as comparators (wRR = 1.03, 95% CI: 0.70–1.53—see Supplementary Table 2).

## Discussion

Overall, delayed puberty was rare while early puberty was common with clear sex differences. Children born to mothers who continued antidepressants in pregnancy or discontinued late in pregnancy had slightly lower risk of early puberty compared to unexposed children or children born to early discontinuers, though confidence intervals were wide and crossed the null. Sex-stratified analyses revealed potentially divergent patterns between male and female offspring but with substantial imprecision. On the other hand, we noted substantial higher crude risk of delayed puberty among children born to continuers but with high level of uncertainty.

Previous epidemiologic studies in humans have linked prenatal antidepressant exposure to subtle alterations in growth trajectories, hormonal regulation, or developmental milestones, which may in turn affect pubertal timing [19–21]. Results from models in rodents have supported this hypothesis. De Oliveira *et al* [5]. suggested a dose-dependent effect of perinatal fluoxetine exposure in delaying testicular maturation in male rat offspring. In addition, Santos *et al* [6]. suggested that maternal fluoxetine administration in rats delayed vaginal opening and first estrus in female offspring, indicating delayed puberty. Crude risks of delayed puberty appeared higher among continuers and late discontinuers; however, these estimates were based on only 17 events and are too imprecise to support firm conclusions. On the other hand, we noted that the onset of puberty has shifted earlier over recent decades in most developed countries and the high proportion of children with early puberty in our cohort is in line with this pattern. In females, earlier menarche is linked to increased lifetime exposure to estrogen, which correlates with a higher risk of breast and endometrial cancer and reduced adult height compared to genetic potential [22]. Early maturation is associated with elevated rates of mental disorders, substance use, and earlier initiation of sexual activity, which can influence educational and socioeconomic trajectories [23]. Our findings, showing no increased risk, or potentially a lower risk, of early puberty among children prenatally exposed to antidepressants, may provide reassurance to women concerned about continuing antidepressant treatment during pregnancy.

The potential sex-specific differences are biologically plausible given that pubertal timing is regulated by separate hormonal pathways in males and females, and that serotonin may interact differently with testosterone versus estrogen signaling [24]. Given limited sample sizes, the observed differences between males and females may reflect random variation rather than true biological heterogeneity. Future work should focus on some complementary areas. First, pooling data from different cohorts would increase the study power, thereby improving the ability to detect small effects, especially for delayed puberty. Second, combining repeated PDS measures with a clinical subsample assessed using Tanner staging alongside hormone panels (e.g. gonadotropins and sex steroids) and growth trajectories would reduce misclassification of outcomes and clarify potential biological pathways.

This study is based on MoBa, one of the largest and most comprehensive prospective birth cohorts worldwide which allowed for exposure data to be collected before the outcome was known, thereby minimizing recall bias. The availability of detailed questionnaires and registry linkages also provided a rich source of covariates, enabling extensive adjustment for potential confounders. From an analytical perspective, the use of GBTM enabled the definition of dynamic exposure patterns, while propensity score weighting ensured adequate balance between exposure groups.

However, several limitations warrant consideration. First, self-reported measures, for both exposure and outcome, introduce a risk of misclassification bias. Moreover, MoBa lacks detailed data on antidepressant dosage and treatment duration making evaluation of dose-response effects impossible. Although we use a large population-based cohort, the number of children that met eligibility criteria and provided complete data on both exposure and outcome was relatively small. Additionally, participation rate in MoBa was approximately 41% with the possibility that women who chose to participate were healthier or otherwise systematically different from non-participants (Supplementary Table 3). Although previous research has suggested that exposure-outcome associations are broadly valid in MoBa, selective participation may affect the generalizability and, potentially, the internal validity of our findings [25]. While propensity score weighting improved covariate balance and minimized some sources of bias, residual confounding due to unmeasured factors such as genetics, family environment or unreported psychiatric severity cannot be ruled out. Lastly, outcome measurements relied on reported PDS rather than clinician Tanner staging. While this approach is pragmatic in large cohorts, some non-differential misclassification is plausible and would bias estimates toward the null. Furthermore, although PDS has demonstrated acceptable validity and reliability in a US adolescent population, it remains a subjective measure and may be influenced by individual perception, social desirability, or limited awareness of pubertal changes. Assessment at a single questionnaire wave may not fully capture the dynamic progression of puberty, potentially leading to misclassification of pubertal timing. Additionally, we lack population-based prevalence of puberty outcomes to assess the potential outcome misclassification, and the measurement properties of this scale in Norwegian population have not been formally established.

## Conclusion

Our findings indicate no increased risk of early puberty following prenatal antidepressant exposure. Delayed puberty was rare but crude estimates suggested higher risk among those exposed during pregnancy. Potentially divergent patterns between male and female offspring might exist and should be investigated further.

## Ethics approval

The current study and linkage with other registries including the Medical Birth Registry of Norway and Norwegian Prescribed Drug Registry were approved by The Regional Committees for Medical and Health Research Ethics on 12 February 2020 (reference number: 63566/REK Sør-Øst), and by the Data Protection Services for research (DPIA number 672954).

## Supplementary Material

dyag240_Supplementary_Data

## Data Availability

Due to Norwegian legislation, the researcher cannot make the data material on an individual level available to others at any time, including in connection with publication. According to the regulations in Norway and the National Institute of Public Health, no MoBa data on an individual or aggregated level may be uploaded into any repository or database at any time. Data from the Norwegian Mother, Father, and Child Cohort Study and the Medical Birth Registry of Norway used in this study are managed by the national health register holders in Norway (Norwegian Institute of Public Health) and can be made available to researchers, provided approval from the Regional Committees for Medical and Health Research Ethics (REC), compliance with the EU General Data Protection Regulation (GDPR) and approval from the data owners. The consent given by the participants does not open for storage of data on an individual level in repositories or journals. Researchers who want access to data sets for replication should apply through helsedata.no.
