# Supplementary material for: Antidepressant use during pregnancy and puberty timing in the offspring: results from the Norwegian Mother, Father, and Child Cohort Study
Source: Int J Epidemiol. 2026 Nov 4;55(6):dyag240. doi: 10.1093/ije/dyag240 (PMC13734381; doi:10.1093/ije/dyag240)
Supplement: Supplementary file 1 [file dyag240_supplementary_data.docx]

**Antidepressant use during pregnancy and puberty timing in the offspring: results from the Norwegian Mother, Father, and Child Cohort Study**

**Authors:** Alessandro Riva, Olav Spigset, Hedvig Nordeng, Per Magnus, Nhung TH Trinh

**APPENDICES**

**Appendix 1. Definition of maternal depression and anxiety disorders**

**Appendix 2. Definition of Pubertal Development Scale**

**Appendix 3. Definition of Obstetric Comorbidity Index**

**Appendix 4. Explanation of the variables used in the study**

**Appendix 5. Selection of the trajectories**

**Figure S1. Directed Acyclic Graph**

**Figure S2. Covariate balance before and after weighting across comparisons**

**Table S1. Count and Risk ratio (RR) of delayed puberty vs normal puberty among offspring across prenatal antidepressant exposure trajectories**

**Table S2. Risk ratio (RR) of early puberty vs normal puberty among offspring across prenatal antidepressant exposure trajectories**

**Table S3. Characteristics of the population that was included or not included in the study population from a subset of singleton pregnancies with mental illnesses before/during/after pregnancy**

**Appendix 1. Definition of maternal depression and anxiety disorders**

Depressive and anxiety disorders were the main underlying conditions to be investigated in this study. These conditions were assessed through several MoBa questionnaires. In Q1, Q3, Q4 and Q-Father they included a list of previous/concurrent illnesses, including depression (Q1 and Q3), anxiety (only Q1) and other mental health disorders (Q1, Q3, Q4 and Q-Father). Women were asked to check off if and when these disorders were present (in Q1 before pregnancy or during early pregnancy, in Q3 during mid-late pregnancy, in Q4 during late pregnancy or after birth and in Q-Father before pregnancy or during early pregnancy) and to report which medication they have taken. Of note, among 16321 singleton pregnancies in our dataset, 7765 pregnancies were identified as having maternal depression/anxiety based on either maternal self-report before pregnancy and/or diagnostic codes for depression/anxiety recorded in the Medical Birth Registry of Norway (MBRN). Among these, 510 (6.6%) were identified from both sources. MBRN identified 28 additional pregnancies with maternal depression/anxiety before pregnancy that were not captured through maternal self-report in the entire dataset and allowed capturing two additional pregnancies in our study population.

The severity of maternal depressive and anxiety symptoms during pregnancy and postpartum was measured via two short versions of The Hopkins Symptom Checklist-25 (SCL-25): the Symptom Checklist-5 (SCL- 5) in Q1 and the Symptom Checklist-8 (SCL-8) in Q3, Q4 and Q-Father. The SCL-25 derived from the SCL-90, which measures several types of symptoms of different mental disorders: symptoms of anxiety with 10 items and of depression with 15 items. The SCL-25 demonstrated a concordance rate of 86.7% between physician assessments and patient self-ratings of psychological distress. The SCL-25 is a widely used self-rating scale designed to measure symptoms of depression and anxiety in population surveys, and it is considered a reliable screening instrument for depression and anxiety according to the ICD-10.

The short version SCL-8 is composed by eight selected items from SCL-25, with four items (1, 2, 7, 8) capturing symptoms of anxiety and four items (3, 4, 5, 6) capturing symptoms of depression. The short version SCL-5 is composed of five selected items of SCL-25, with two items (1, 2) capturing symptoms of anxiety and three items (3, 4, 5) capturing symptoms of depression. Response categories are the same for all items: "Not bothered", "A little bothered", "Quite bothered" and "Very bothered," rated from 1 to 4 respectively. The total sum score was calculated for SCL-5 and SCL-8 separately and then divided by the number of items in each instrument. In both SCL-5 and SCL-8, a mean score of greater than 2.0 was defined as indicative of the presence of depression.

The SCL-5 has shown a high correlation (r = 0.92) with the total score of the original SCL-25, as well as good internal consistency (Cronbach’s alpha = 0.85). Its diagnostic performance has also been evaluated, with sensitivity and specificity estimated at 82% and 96%, respectively. The SCL-8 has demonstrated a high correlation (r = 0.94) with the total score of the original SCL-25. Additionally, its anxiety and depression subscales have shown strong correlations with the corresponding subscales of the original instrument (r = 0.90 and r = 0.92, respectively). The internal consistency (Cronbach’s alpha) of the SCL-8 has been estimated at 0.88 for the total score, 0.78 for the anxiety subscale and 0.82 for the depression subscale.

Postnatal depression was assessed in Q4 using the Edinburgh Postnatal Depression Scale (EPDS), a 10-item self-report questionnaire developed by Cox *et al*. (1987) in primary care settings in Livingston and Edinburgh. The EPDS was designed to help healthcare professionals identify mothers experiencing symptoms of postnatal depression. It incorporates items adapted from existing scales such as the Depression and Anxiety Scale (IDA), the Hospital Anxiety and Depression Scale (HAD), the Anxiety and Depression Scale by Bedford and Foulds, as well as items developed specifically for the scale. Of the ten items, five assess dysphoric mood, two assess anxiety and one each relates to guilt, suicidal ideation and coping difficulties. Each item is scored from 0 to 4, with response options ranging from “No, never” to “Yes, most of the time” and higher scores indicating greater severity of depressive symptoms.

A shorter version of the EPDS, the EPDS-5, was validated by Eberhard-Gran *et al*. (2007) and adopted in the MoBa study. The five selected items excluded those assessing dysphoric mood to avoid potential confounding with somatic symptoms related to childbirth. However, due to a miscommunication during implementation in MoBa, one of the five intended items (“I have looked forward with enjoyment to things”) was mistakenly replaced with two alternative EPDS items (items 3 and 6). An adapted scoring method was applied and a total score greater than 8 was used as the threshold to indicate probable postnatal depression. Test reliability estimates range from 0.82 to 0.88, while internal consistency (Cronbach’s alpha) has been reported between 0.77 and 0.88. This short version of the scale demonstrated a high correlation with the full scale (r = 0.95) and an alpha reliability of 0.80.

LifeTime History (LTH) of Major Depression (MD) was assessed in Q1 through self-reported responses to five key depressive symptoms, selected from the nine diagnostic criteria for Major Depression in the Diagnostic and Statistical Manual of Mental Disorders, Third Edition Revised (DSM-III-R). Each symptom was answered with “yes” or “no”. These items closely align with DSM-III criteria for identifying lifetime major depression.

A case of LTH of MD was considered present when the following conditions were met:

- The respondent reported experiencing at least three distinct types of depressive symptoms
- One of these symptoms was the first, “Felt depressed, sad”
- Three types of symptoms occurred simultaneously

The criteria also include that depression was not triggered by an identifiable negative life event.

The reliability of this diagnostic approach was evaluated in a follow-up study conducted one year later, in which participants completed the Composite International Diagnostic Interview (CIDI), a structured diagnostic instrument. The agreement between the self-reported symptom-based scale and the CIDI diagnosis was modest, with a kappa coefficient of 0.34 and a tetrachoric correlation of 0.56.

**Appendix 2. Definition of Pubertal Development Scale**

In the 14-years questionnaire, mothers reported their children's pubertal status using the Self-rating Scale for Pubertal Development Scale (PDS). The PDS is an adaptation of an interview-based puberty-rating scale originally developed by Petersen *et al*., and serves as a useful tool for assessing pubertal development in large-scale studies requiring non-invasive measures.

The validity of the PDS was supported by Carskadon *et al*. (1993). PDS was first evaluated in a sample including 17 girls (ages 10-16 years) and 21 boys (ages 9-16 years) who were also examined by a pediatrician for conventional Tanner staging. The pediatrician was blind to the subjects’ self-evaluations. Mean self-reporting scores from the children were compared to the pediatrician’s assessment of Tanner stage based upon public hair growth. The Spearman correlation coefficient between self-reported and pediatrician-rated physical development was high (r=0.868-0.841, p<0.001) and a high correspondence between the two measures of physical development was indicated). Only 7 of the 38 adolescents rated themselves as much as one stage different from the pediatrician. Six of these discordant ratings were from boys. Then PDS was further evaluated in a sample of 698 5th- and 6th-grade students (323 boys and 375 girls), along with reports from their parents and teachers. Study participants comprised of students from 61 schools across 31 U.S. states, the majority of whom were Caucasian (87%) and living in smaller population areas (<50,000 inhabitants). They found significant correlations between parent and student ratings for all pubertal measures in 6th-graders and 5th-grade girls, and for several measures in 5th-grade boys. The findings showed that 5th-grade students rated themselves, and were rated by their parents, as less developmentally mature than 6th-graders. Additionally, 6th-grade girls were consistently rated as more mature than boys of the same age, in line with typical pubertal trajectories. Although the Tanner staging method via clinical examination remains the gold standard for evaluating pubertal status, the PDS has shown acceptable validity when compared to physician assessments. Regarding reliability, internal consistency of the PDS was reported as adequate in the original U.S.-based validation study, with Cronbach’s alpha values ranging from 0.67 to 0.70 for the student version and from 0.68 to 0.78 for the parent version, indicating reasonably consistent responses (Carskadon & Acebo, 1993). However, these reliability estimates were derived from a U.S. school-based population and may not directly generalize to a Norwegian setting. Its measurement properties in Norwegian populations have not been formally established.

The scale assesses general markers of pubertal development, including growth spurt (length), body hair growth and skin changes (pimples), as well as gender-specific characteristics such as voice deepening and facial hair for boys, and breast development and menstruation for girls. Each item is rated using the following response options, which are assigned point values: “Not yet started” = 1 point, “Barely started” = 2 points, “Definitely started” = 3 points, “Already complete” = 4 points, For menstruation “Yes” = 4 points and “No” = 1 point.

- For boys, PDS score is the average of the point values for questions on body growth, body hair growth, skin changes, voice changes and facial hair growth:

$${PDS score}_{boys}=\frac{UB222+UB223+UB224+UB227+UB228}{5}$$

- For girls, PDS score is the average of the point values for questions on body growth, body hair growth, skin changes and breast development:

$${PDS score}_{girls}=\frac{UB222+UB223+UB224+UB229+UB230}{5}$$

To align with Tanner stages, Crocket, as reported by Carskadon and Acebo, proposed a 5-point categorical classification of pubertal development based on PDS scores:

| Male | Female |
| --- | --- |
| Pre-pubertal: PDS score ≤ 1.5 | Pre-pubertal: PDS score ≤ 1.7 |
| Early pubertal: PDS score > 1.5 - ≤ 2.0 | Early pubertal: PDS score > 1.7 - ≤ 2.2 |
| Mid-pubertal: PDS score > 2.0 - ≤ 2.5 | Mid-pubertal: PDS score > 2.2 - ≤ 2.7 |
| Late pubertal: PDS score > 2.5 - ≤ 3.0 | Late pubertal: PDS score > 2.7 - ≤ 3.2 |
| Post-pubertal: PDS score > 3.0 | Post-pubertal: PDS score > 3.2 |

**Appendix 3. Definition of Obstetric Comorbidity Index**

The Obstetric Comorbidity Index, developed by Bateman *et al*. in 2013, is a validated scoring system designed to assess the cumulative burden of preexisting medical conditions in pregnant women at the time of delivery admission and predict the risk of severe maternal morbidity (SMM). SMM encompasses life-threatening complications such as end-organ damage, Intensive Care Unit (ICU) admission and maternal death. The index incorporates 20 maternal comorbidities and maternal age, each assigned an integer weight based on its adjusted Odds Ratio (aOR) for SMM, derived from multivariable logistic regression. These aORs were rounded to the nearest multiple of five and transformed into standardized weights to ensure clinical utility. The total OCI score is calculated by summing the weights of all comorbidities present in an individual, with scores ranging from 0 (no comorbidities) to a theoretical maximum of 48. Critically, higher OCI scores demonstrate a dose-dependent relationship with SMM risk, enabling clinicians to stratify patients for targeted monitoring and intervention.

| Condition: | Weights: |
| --- | --- |
| Severe preeclampsia/eclampsia | 5 |
| Chronic congestive heart failure | 5 |
| Congenital heart disease | 4 |
| Pulmonary hypertension | 4 |
| Chronic ischemic heart disease | 3 |
| Sickle cell disease | 3 |
| Multiple gestation | 2 |
| Cardiac valvular disease | 2 |
| Systemic lupus erythematosus | 2 |
| Human immunodeficiency virus | 2 |
| Mild or unspecified preeclampsia (only if they did not have severe preeclampsia/eclampsia | 2 |
| Drug abuse | 2 |
| Placenta previa | 2 |
| Chronic renal disease | 1 |
| Pre-existing hypertension | 1 |
| Previous cesarean delivery | 1 |
| Gestational hypertension | 1 |
| Alcohol abuse | 1 |
| Asthma | 1 |
| Pre-existing diabetes mellitus | 1 |
| Maternal age – years | Weights: |
| > 44 | 3 |
| 40 – 44 | 2 |
| 35 – 39 | 1 |

In this study, the OCI was calculated using data from the MoBa Q1 questionnaire and the MBRN dataset. Due to available data, several adaptations were necessary. First, multiple gestation was excluded in the calculation, as the study sample consisted exclusively of singleton children. Second, pulmonary hypertension, Human Immunodeficiency Virus (HIV), placenta previa and previous cesarean delivery were omitted because relevant data were unavailable. Third, for certain conditions where a specific variable indicating the presence of the disease was not available, similar or closely related variables were used to identify their presence. Fourth, chronic congestive heart failure, chronic ischemic heart disease and cardiac valvular disease were grouped and considered as a single condition, as their definitions overlapped in the available data. These three cardiovascular conditions were assigned with a combined weight of 3, corresponding to the average of their original weights (5, 3, and 2). As a result of these modifications, the OCI score in this study ranges from 0 to 30, rather than the original 0 to 48. This adjusted range reflects the exclusion of multiple gestation (original weight 2) and pulmonary hypertension (original weight 4), HIV (original weight 2), previous cesarean delivery (original weight 1), placenta previa (original weight 2), the grouping of the three cardiovascular conditions (total original weight 10, replaced with a weight of 3) and the retention of all other comorbidities as defined in the original index.

**Appendix 4. Explanation of the variables used in the study**

| Variable | Explanation | Data Source |
| --- | --- | --- |
| Maternal variables | | |
| Mother age at delivery  - Number | Mother`s age at the time of birth in years | MBRN |
| Parity  - Primiparous  - Multiparous | - Primiparous: number of previous deliveries and number of previous miscarriages or stillbirths from week 12 of pregnancy = 0  - Multiparous: number of previous deliveries = 1 or more | MBRN |
| History of abortions and miscarriages  - Yes  - No | - Yes: number of previous miscarriages or stillbirths from week 12 of pregnancy = 1 or more  - No: number of previous miscarriages or stillbirths from week 12 of pregnancy = 0 | MBRN |
| Length of gestation  - Number | Number of completed weeks, based on ultrasound estimations. If ultrasound is not available, the gestational length is calculated from the last menstrual period | MBRN |
| Prematurity  - Yes  - No | - Yes: number of completed weeks $<$ 37  - No: number of completed weeks $\geq$ 37 | MBRN |
| Body Mass Index (BMI) at conception  - Number | In MoBA Q1, respondents were asked about their height in centimeter and their weight when they became pregnant in kilograms  🡪 $BMI=\frac{{Weight}_{Conception}}{\left( \frac{Height}{100} \right)^{2}}$ | MoBa |
| Body Mass Index (BMI) at conception categorized  - Underweight  - Normal Weight  - Overweight  - Obese | - Underweight: BMI $<$18.5  - Normal Weight: BMI $\geq$18.5 and <25  - Overweight: BMI $\geq$25 and <30  - Overweight: BMI $\geq$30 | MoBa |
| Weight gain during pregnancy | In MoBa Q1 and Q4, respondents were asked their weight at start and at the end of pregnancy in kilograms  🡪$Weight Gain={Weight}_{Delivery}-{Weight}_{Conception}$ | MoBa |
| Weight gain during pregnancy categorized  - Too little  - Adequate  - Too much | - Too little: Underweight BMI and pregnancy weight gain <12.5, Normal Weight BMI and pregnancy weight gain <11.5, Overweight BMI and pregnancy weight gain <7, Overweight BMI and pregnancy weight gain <5  - Adequate: Underweight BMI and pregnancy weight gain $\geq$12.5 and $\leq$18, Normal Weight BMI and pregnancy weight gain $\geq$11.5 and $\leq$16, Overweight BMI and pregnancy weight gain $\geq$7 and $\leq$11.5, Overweight BMI and pregnancy weight gain $\geq$5 and $\leq$9  - Too much: Underweight BMI and pregnancy weight gain $>$18, Normal Weight BMI and pregnancy weight gain $>$16, Overweight BMI and pregnancy weight gain $>$11.5, Overweight BMI and pregnancy weight gain >9 | MoBa |
| Sleeping problems during pregnancy  - Yes  - No | In MoBa Q1, respondents were asked if and when they had experienced any illnesses or problems during this pregnancy  - Yes: answered in the line of “Unusual tiredness/sleepiness” and/or of “Sleeping problems”  - No: not answered in the line of “Unusual tiredness/sleepiness” and of “Sleeping problems” | MoBa |
| Smoking before pregnancy  - Yes  - No | In MoBa Q1, respondents were asked if they had smoked in the last 3 months before they became pregnant. If sometimes, respondents were also asked how many cigarettes per week; if daily, respondents were also asked how many cigarettes per day  - Yes: answered “Daily” or “Sometimes” or more than 0 in the second question  - No: answered “No” and 0 or missing in the second question | MoBa |
| Smoking during pregnancy  - Yes  - No | In MoBa Q1 and Q3, respondents were asked if they had smoked during this pregnancy. If sometimes, respondents were also asked how many cigarettes per week; if daily, respondents were also asked how many cigarettes per day  - Yes: answered “Daily” or “Sometimes” or more than 0 in the second question  - No: answered “No” and 0 or missing in the second question | MoBa |
| Smoking after childbirth  - Yes  - No | In MoBa Q4, respondents were asked if they had smoked after childbirth. If smoked sometimes, respondents were also asked how many cigarettes per week; if smoked every day, respondents were also asked how many cigarettes per day  - Yes: answered “Smoked sometimes” or “Smoked every day” after birth or more than 0 in the second question  - No: answered “Didn’t smoke” after the birth and 0 or missing in the second question | MoBa |
| Nicotine use before pregnancy  - Yes  - No | In MoBa Q1, respondents were asked if they had used other kinds of nicotine and indicate which and when they used them  - Yes: answered “Before pregnancy”  - No: not answered “Before pregnancy” | MoBa |
| Nicotine use during pregnancy  - Yes  - No | In MoBa Q1 and Q3, respondents were asked if they had used other kinds of nicotine and indicate which and when they used them  - Yes: answered “After pregnancy” in Q1 or answered “Yes” in Q3  - No: not answered “After pregnancy” in Q1 and answered “No” in Q3. | MoBa |
| Alcohol consumption before pregnancy  - Yes  - No | In MoBa Q1, respondents were asked how often they consumed alcohol in the 3 months before they became pregnant  - Yes: answered “Approximately 6-7 time a week” or “Approximately 4-5 times a week” or “Approximately 2-3 times a week” or “Approximately once a week”  - No: answered “Approximately 1-3 times a month” or “Less than once a month” or “Never” | MoBa |
| Alcohol consumption during pregnancy  - Yes  - No | In MoBa Q1, Q3 and Q4, respondents were asked how often they consumed alcohol during this pregnancy  - Yes: answered “Approximately 6-7 time a week” or “Approximately 4-5 times a week” or “Approximately 2-3 times a week” or “Approximately once a week in Q1 or answered “Roughly 6-7 time a week” or “Roughly 4-5 times a week” or “Roughly 2-3 times a week” or “Roughly once a week in Q3 and Q4  - No: not answered “Approximately 1-3 times a month” or “Less than once a month” or “Never” in Q1 and answered “Roughly 1-3 times a month” or “Less than once a month” or “Never” in Q3 | MoBa |
| Alcohol consumption after childbirth  - Yes  - No | In MoBa Q4, respondents were asked how often they consumed alcohol after childbirth  Yes: answered “Roughly 6-7 time a week” or “Roughly 4-5 times a week” or “Roughly 2-3 times a week” or “Roughly once a week  - No: answered “Roughly 1-3 times a month” or “Less than once a month” or “Never” | MoBa |
| Illicit substance use before pregnancy  - Yes  - No | In MoBa Q1, respondents were asked if they had ever used an illicit substance (Hash, Amphetamine, Ecstasy, Cocaine, Heroin, Anabolic steroids, Testosterone products, Growth hormones) before they became pregnant  - Yes: answered in “Last months before pregnancy”  - No: not answered in “Last months before pregnancy” | MoBa |
| Illicit substance use during pregnancy  - Yes  - No | In MoBa Q1, Q3 and Q4, respondents were asked if they had ever used an illicit substance (Hash, Amphetamine, Ecstasy, Cocaine, Heroin, Anabolic steroids, Testosterone products, Growth hormones) during this pregnancy  - Yes: answered in “During pregnancy” in Q1 or answered “Yes” in Q3  - No: not answered in “During pregnancy” in Q1 and answered “No” in Q3 | MoBa |
| Illicit substance use after pregnancy  - Yes  - No | In MoBa Q4, respondents were asked if they had ever used an illicit substance (Hash, Amphetamine, Ecstasy, Cocaine, Heroin, Anabolic steroids, Testosterone products, Growth hormones) after childbirth  - Yes: answered “Yes, after birth”  - No: answered “No” | MoBa |
| Folate supplements before pregnancy  - Yes  - No | In MoBa Q1, respondents were asked if they had taken any vitamins, minerals or other nutritional supplements before pregnancy. If yes, they were asked to fill a table below for the vitamins and minerals found in the list of ingredients on the vitamin package/bottle  - Yes: answered in the line of “Folic acid”  - No: not answered in the line of “Folic acid” | MoBa |
| Folate supplements during pregnancy  - Yes  - No | In MoBa Q1 and Q3, respondents were asked if they had taken any vitamins, minerals or other nutritional supplements during this pregnancy. If yes, they were asked to fill a table below for the vitamins and minerals found in the list of ingredients on the vitamin package/bottle  - Yes: answered in the line of “Folic acid”  - No: not answered in the line of “Folic acid” | MoBa |
| Self-reported depression before pregnancy  - Yes  - No | In MoBa Q1, respondents were asked if and when they had experienced any illnesses or problems before pregnancy  - Yes: answered in the line of “Depression”  - No: not answered in the line of “Depression” | MoBa |
| Self-reported depression during pregnancy  - Yes  - No | In MoBa Q1 and Q3, respondents were asked if and when they had experienced any illnesses or problems during this pregnancy  - Yes: answered in the line of “Depression”  - No: not answered in the line of “Depression” | MoBa |
| Self-reported anxiety before pregnancy  - Yes  - No | In MoBa Q1, respondents were asked if and when they had experienced any illnesses or problems before pregnancy  - Yes: answered in the line of “Anxiety”  - No: not answered in the line of “Anxiety” | MoBa |
| Self-reported anxiety during pregnancy  - Yes  - No | In MoBa Q1, respondents were asked if and when they had experienced any illnesses or problems during this pregnancy  - Yes: answered in the line of “Depression”  - No: not answered in the line of “Depression” | MoBa |
| Self-reported mental health problems after childbirth  - Yes  - No | In MoBa Q4, respondents were asked if and when they had experienced any illnesses or problems during this pregnancy  - Yes: answered in the line of “Mental health problems”  - No: not answered in the line of “Mental health problems” | MoBa |
| Depression and anxiety during pregnancy according to Symptom Checklist-5 (SCL- 5) and Symptom Checklist-8 (SCL-8)  - $\geq$2.0  - $<$ 2.0 | In MoBa Q1 and Q3, respondents were asked if they had been bothered by any of the problems listed in the table below during the last two weeks  - $\geq$2.0: if the mean score calculated for SCL-5 and SCL-8 is $\geq$ 2.0  - $<$ 2.0: if the mean score calculated for SCL-5 and/or SCL-8 is $<$ 2.0  See the Materials and Methods section for more details | MoBa |
| Depression and anxiety after childbirth according to Symptom Checklist-8 (SCL-8)  - $\geq$2.0  - $<$ 2.0 | In MoBa Q4, respondents were asked if they had been bothered by any of the problems listed in the table below during the last two weeks  - $\geq$2.0: if the mean score calculated for SCL-8 is $\geq$ 2.0  - $<$ 2.0: if the mean score calculated for SCL-8 is $<$2.0  See the Materials and Methods section for more details | MoBa |
| Postnatal depression according to Edinburgh Postnatal Depression Scale (EPDS-5)  - Yes  - No | In MoBa Q4, respondents were asked if they had been experienced any of the feelings listed in the table below during the last two weeks  - Yes: if the total score is $>$ 8 and no SCL or self-reported depression  - No: if the total score is $\leq$8 or if SCL depression or if self-reported depression  See the Materials and Methods section for more details | MoBa |
| Lifetime History (LTH) of Major Depression (MD) according to criteria for Major Depression in the Diagnostic and Statistical Manual of Mental Disorders (DSM-lll-R)  - Yes  - No | In MoBa Q1, respondents were asked if they had ever experienced any of the problems listed in the table below for a period of 2 weeks or more in their life  - Yes: if they answered “Yes” in three or more symptoms, answered “yes” in “Felt depressed, sad” and answered “Yes” in “Had at least 3 of the problems named above simultaneously”  - No: if they answered “Yes” in two or less symptoms, answered “No” in “Felt depressed, sad” and answered “No” in “Had at least 3 of the problems named above simultaneously”  See the “Materials and Methods” section for more details | MoBa |
| Co-medication with opioids (ATC code N02A), benzodiazedine derivates (ATC code N05BA), hypnotics and sedatives (ATC code N05C), antipsychotics (ATC code N05A) and antiepileptics (ATC code N03A) before pregnancy  - Yes  - No | In MoBa Q1, respondents were asked if and when they had experienced any illnesses or problems before pregnancy, and if and when they had taken any medication for the illnesses or problems they had responded to  In MoBa Q1, respondents were also asked if and when they had taken other medication not previously mentioned before pregnancy  - Yes: medication taken belongs to one or more of the following groups: opioids (ATC code N02A), benzodiazedine derivates (ATC code N05BA), hypnotics and sedatives (ATC code N05C), antipsychotics (ATC code N05A) and antiepileptics (ATC code N03A)  - No: no medication taken belongs to one or more of the following groups: opioids (ATC code N02A), benzodiazedine derivates (ATC code N05BA), hypnotics and sedatives (ATC code N05C), antipsychotics (ATC code N05A) and antiepileptics (ATC code N03A). | MoBa |
| Co-medication with opioids (ATC code N02A), benzodiazedine derivates (ATC code N05BA), hypnotics and sedatives (ATC code N05C), antipsychotics (ATC code N05A) and antiepileptics (ATC code N03A) during pregnancy  - Yes  - No | Respondents were asked if and when they had experienced any illnesses or problems during this pregnancy, and if and when they had taken any medication for the illnesses or problems they had responded to  In MoBa Q1 and Q3, respondents were also asked if and when they had taken other medication not previously mentioned during this pregnancy:  - Yes: medication taken belongs to one or more of the following groups: opioids (ATC code N02A), benzodiazedine derivates (ATC code N05BA), hypnotics and sedatives (ATC code N05C), antipsychotics (ATC code N05A) and antiepileptics (ATC code N03A)  - No: no medication taken belongs to one or more of the following groups: opioids (ATC code N02A), benzodiazedine derivates (ATC code N05BA), hypnotics and sedatives (ATC code N05C), antipsychotics (ATC code N05A) and antiepileptics (ATC code N03A) | MoBa |
| Co-medication with opioids (ATC code N02A), benzodiazedine derivates (ATC code N05BA), hypnotics and sedatives (ATC code N05C), antipsychotics (ATC code N05A) and antiepileptics (ATC code N03A) after childbirth  - Yes  - No | In MoBa Q4, respondents were asked if and when they had experienced any illnesses or problems after childbirth, and if and when they had taken any medication for the illnesses or problems they had responded to  In MoBa Q4, respondents were also asked if and when they had taken other medication not previously mentioned after childbirth  - Yes: medication taken belongs to one or more of the following groups: opioids (ATC code N02A), benzodiazedine derivates (ATC code N05BA), hypnotics and sedatives (ATC code N05C), antipsychotics (ATC code N05A) and antiepileptics (ATC code N03A)  - No: no medication taken belongs to one or more of the following groups: opioids (ATC code N02A), benzodiazedine derivates (ATC code N05BA), hypnotics and sedatives (ATC code N05C), antipsychotics (ATC code N05A) and antiepileptics (ATC code N03A) | MoBa |
| Obstetric Comorbity Index (OCI) categorized  - 0  - 1-3  - 4-6  - $>$6 | – 0: OCI = 0  - 1-3: OCI $\geq$1 and $\leq$3  - 4-6: OCI $\geq$4 and $\leq$6  - >6: OCI $\geq$7 |  |
|  | - Severe preeclampsia/eclampsia: Preeclampsia (2 = Serious)  - Mild or unspecified preeclampsia: Preeclampsia (1 = Light, 3 = Unspecified)  - Chronic renal disease: Chronic renal disease (1 = Yes, 0 = No)  - Pre-existing hypertension: Chronic hypertension prior to pregnancy (1 = Yes, 0 = No)  - Gestational hypertension: Hypertensive only during pregnancy (1 = Yes, 0 = No)  - Asthma: Asthma (1 = Yes, 0 = No)  - Pre-existing diabetes mellitus: Pregestational diabetes (1 = Type 1, 2 = Type 2, 3 = Unspecified / Other)  - Maternal age, Years: 🡪 Mother`s age (In years at the time of Birth)  See the “Materials and Methods” section for more details | MBRN |
|  | - Congenital heart disease: In MoBa Q1, respondents were asked if and when they had experienced any illnesses or problems before and during pregnancy: answered in the line “Congenital heart defect”  - Heart diseases: In MoBa Q1, respondents were asked if and when they had experienced any illnesses or problems before and during pregnancy: answered in the line “Other heart disease”  - Sickle cell disease: In MoBa Q1, respondents were asked if and when they had experienced any illnesses or problems before and during pregnancy: answered in the line “Anaemia/low hemoglobin”  - Systemic lupus erythematosus: In MoBa Q1, respondents were asked if and when they had experienced any illnesses or problems before and during pregnancy: answered in the line “Lupus (SLE)”  - Drug abuse: In MoBa Q1, respondents were asked if they had ever used an illicit substance (Hash, Amphetamine, Ecstasy, Cocaine, Heroin) before and during pregnancy: answered “Last months before pregnancy” and/or “During pregnancy”  - Alcohol abuse: In MoBa Q1, respondents were asked how often they consume alcohol in the 3 months before and during pregnancy: answered “Approximately 6-7 time a week” or “Approximately 4-5 times a week”  See the “Materials and Methods” section for more details | MoBa |
| Paternal variables | | |
| Father age at delivery  - Number | Father`s age at the time of birth in years | MBRN |
| Depression and anxiety during pregnancy according to Symptom Checklist-8 (SCL-8)  - $\geq$2.0  - $<$ 2.0 | In MoBa Q-Father, respondents were asked if they had been bothered by any of the problems listed in the table below during the last two weeks  - $\geq$2.0: if the mean score calculated for SCL-8 is $\geq$2.0  - $<$ 2.0  : if the mean score calculated for SCL-8 is $<$ 2.0  See the Materials and Methods section for more details | MoBa |
| Lifetime History (LTH) of Major Depression (MD) measured according to criteria for Major Depression in the Diagnostic and Statistical Manual of Mental Disorders (DSM-lll-R)  - Yes  - No | In MoBa Q-Father, respondents were asked if they had ever experienced any of the problems listed in the table below for a period of 2 weeks or more in their life  - Yes: if they answered “Yes” in three or more symptoms, answered “yes” in “Felt depressed, sad” and answered “Yes” in “Had at least 3 of the problems named above simultaneously”  - No: if they answered “Yes” in two or less symptoms, answered “No” in “Felt depressed, sad” and answered “No” in “Had at least 3 of the problems named above simultaneously”  See the “Materials and Methods” section for more details | MoBa |
| Child variables | | |
| Child sex  - Male  - Female | Sex as reported:  - Male = 1  - Female = 2 | MBRN |
| Child weight at birth  - Number | Child’s weight in grams | MBRN |
| Child weight categorized  - Low  - Normal  - High | - Low: $<$2500 grams  - Normal: $\geq$2500 and $<$4500  - High: $\geq$4500 | MBRN |
| Child length at birth  - Number | Child’s length in cm | MBRN |
| Gestation age  - Number | Length of gestation in days, based on ultrasound estimations. If ultrasound is not available, the gestational length is calculated from the last menstrual period | MBRN |
| Breastfeeding  - From 1 month to 7 months  - No | In MoBa Q4, respondents were asked what they had given to the child to drink during the first week of life and during the first 6 months of his/her life and how often they give their child the following drink at the moment  - From 1 month to 7 months: answered in the line of “Breast milk” in first question, answered in the line of “Breast milk” in the second question and answered in the line of “Breast milk” or “Break milk supplement” “4-6 times a week” or “At least once a day” or “1-3 times a week” in the third question  - No: not answered in the line of “Breast milk” in first question | MoBa |
| Socioeconomic variables | | |
| Maternal civil status  - Married  - Co-habitant  - Unmarried/Single  - Divorced/ Separated/Widow | - Married as reported: 1 = Married  - Co-habitant as reported: 2 = Co-habitant  - Unmarried/Single as reported: 3 = Unmarried/Single  - Divorced/ Separated/Widow as reported: 4 = Divorced/ Separated/Widow | MBRN |
| Mother income  - None  - Low  - Average  - High | In MoBa Q1, respondents were asked about their mother’s and father’s yearly gross income  - None: no income  - Low: total gross annual income of mother $\leq$199.999 NOK  - Average: total gross annual income of mother $\geq$200.000 NOK and $\leq$499.999 NOK  - High: total gross annual income of mother $\geq$500.000 NOK | MoBa |
| Father income  - None  - Low  - Average  - High | In MoBa Q1, respondents were asked about their mother’s and father’s yearly gross income  - None: no income  - Low: total gross annual income of father $\leq$199.999 NOK  - Average: total gross annual income of father $\geq$200.000 NOK and $\leq$499.999 NOK  - High: total gross annual income of father $\geq$500.000 NOK | MoBa |
| Mother education level  - Secondary school  - High school  - University | In MoBa Q1, respondents were asked about their education level  - Secondary school: answered “9-year secondary school”  - High school: answered “1-2 year high school” or “Vocational high school” or “3-year high school general studies, junior college”  - University: answered “Regional technical college, 4-year University degree (Bachelor’s degree, nurse, teacher, engineer)” or “University, technical college, more than 4 years (Master’s degree, medical doctor, PhD)” | MoBa |
| Father educational level  - Secondary school  - High school  - University or college | In MoBa Q1 and Q-Father, respondents were asked about their education level  - Secondary school: answered “9-year secondary school” in Q1 or answered “Secondary school” in Q-Father  - High school: answered “1-2 year high school” or “Vocational high school” or “3-year high school general studies, junior college” in Q1 or answered “Further education 1-2 years” or “Further education-vocational” or “Further education 3 years (general studies, sixth form)” in Q-Father  - University or college: answered “Regional technical college, 4-year university degree (Bachelor’s degree, nurse, teacher, engineer)” or “University, technical college, more than 4 years (Master’s degree, medical doctor, PhD)” in Q1 or answered “Higher education (University/college), up to and including 4 years” or “Higher education (University/college), over 4 years” in Q-Father | MoBa |

**Appendix 5. Selection of the trajectories**

| Model | BIC | AIC | Entropy | Group 1 | Group 2 | Group 3 | Group 4 | Group 5 |
| --- | --- | --- | --- | --- | --- | --- | --- | --- |
| Two trajectories with linear functions (1 1) - A | -759.87 | -745.68 | 0.980 | 69.95% | 30.05% |  |  |  |
| Three trajectories with linear functions (1 1 1) – B | -680.50 | -657.79 | 0.886 | 22.56% | 55.78% | 21.66% |  |  |
| Four trajectories with linear functions (1 1 1 1) – C | -681.46 | -650.23 | 0.988 | 22.50% | 10.40% | 55.79% | 11.31% |  |
| Five trajectories with linear functions (1 1 1 1 1) - D | -674.43 | -634.69 | 0.999 | 7.56% | 25.33% | 48.04%, | 4.83% | 14.23% |
| Three trajectories with quadratic functions (2 2 2) | -703.03 | -671.81 | 0.901 | 22.56% | 55.78% | 21.66% |  |  |
| Three trajectories with cubic functions (3 3 3) | -678.78 | -639.04 | 0.908 | 22.56% | 55.78% | 21.66% |  |  |

|   A |   B |
| --- | --- |
|   C |   D |

Model with two trajectories (1,1): this model has lowest BIC and highest entropy but does not capture different discontinuation timings.

Model with three trajectories (1,1,1): This model performs slightly worse than the two-trajectory model in terms of BIC and entropy. However, it separates two distinct discontinuation patterns which are expected from the clinical point of view.

Model with four trajectories (1,1,1,1): This model performs slightly worse than the two-trajectory model in term of BIC and has comparable entropy. However, the groups become smaller (10%) when the two continuations patterns are separated.

Model with five trajectories (1,1,1,1,1): this model was over-complex and yielded very small sub-groups (less than 5%).

Given no major differences in the statistical criteria across the models, the model with three trajectories was then selected by balancing performance and clinical interpretation. We then increased the complexity by increasing the order of the polynomial functions. The final model included quadratic time functions.

**Figure S1. Directed Acyclic Graphs**

**Figure S2. Covariate balance before and after weighting across comparisons (average standardized mean differences across imputations)**

Continuers vs early discontinuers

Continuers vs unexposed

Early discontinuers vs unexposed

Late discontinuers vs early discontinuers

Late discontinuers vs unexposed

Exposed vs unexposed

Exposed vs early discontinuers

**Table S1. Count and Risk ratio (RR) of delayed puberty vs normal puberty among offspring across prenatal antidepressant exposure trajectories**

| **Comparison** | **Event rate** | **Crude RR** |
| --- | --- | --- |
| Continuers vs Unexposed | <5/31 vs 13/618 | 1.53 (0.21, 11.37) |
| Early discontinuers vs unexposed | <5/82 vs 13/618 | 0.58 (0.08, 4.38) |
| Late discontinuers vs unexposed | <5/34 vs 13/618 | 2.80 (0.66, 11.91) |
| Continuers vs early discontinuers | <5/31 vs <5/82 | 2.65 (0.17, 41.50) |
| Late vs early discontinuers | <5/34 vs <5/82 | 4.82 (0.45, 51.97) |

**Table S2. Risk ratio (RR) of early puberty vs normal puberty among offspring across prenatal antidepressant exposure trajectories**

| **Comparison** | **Event rates** | **Unweighted relative risk** | **Weighted relative risk** |
| --- | --- | --- | --- |
| **Overall** | | | |
| Exposed vs unexposed | 35/100 vs 538/1156 | 0.77 (0.58, 1.01) | 0.80 (0.60, 1.07) |
| Exposed vs early discontinuers | 35/100 vs 58/140 | 0.86 (0.62, 1.20) | 1.03 (0.70, 1.53) |
| **Female** | | | |
| Exposed vs unexposed | 26/56 vs 371/608 | 0.76 (0.57, 1.02) | 0.82 (0.57, 1.18) |
| Exposed vs early discontinuers | 26/56 vs 37/66 | 0.82 (0.57, 1.16) | 0.95 (0.58, 1.56) |
| **Male** | | | |
| Exposed vs unexposed | 9/44 vs 167/548 | 0.70 (0.39, 1.27) | 0.75 (0.40, 1.41) |
| Exposed vs early discontinuers | 9/44 vs 21/74 | 0.77 (0.39, 1.53) | 1.07 (0.50, 2.28) |

**Table S3. Characteristics of the population that was included or not included in the study population from a subset of singleton pregnancies with mental illnesses before/during/after pregnancy**

|  | Not included in the study population | Included in the study population | Total |
| --- | --- | --- | --- |
| N | 14,925 (91.4%) | 1,396 (8.6%) | 16,321 (100.0%) |
| Maternal age (years, mean (SD)) | 29.8 (4.9) | 30.7 (4.8) | 29.9 (4.9) |
| Maternal age categories |  |  |  |
| *<25 years* | 2,166 (14.5%) | 143 (10.2%) | 2,309 (14.1%) |
| *25-35 years* | 10,905 (73.1%) | 1,038 (74.4%) | 11,943 (73.2%) |
| *>=36* | 1,854 (12.4%) | 215 (15.4%) | 2,069 (12.7%) |
| Parity |  |  |  |
| *Primiparous* | 7,007 (46.9%) | 733 (52.5%) | 7,740 (47.4%) |
| *Multiparous* | 7,918 (53.1%) | 663 (47.5%) | 8,581 (52.6%) |
| Previous miscarriages or abortions | 3,247 (24.7%) | 303 (25.7%) | 3,550 (24.8%) |
| Length of gestation in weeks | 39.5 (1.5) | 39.6 (1.6) | 39.5 (1.5) |
| Prematurity | 862 (5.8%) | 71 (5.1%) | 933 (5.7%) |
| Maternal BMI at conception |  |  |  |
| *Underweight* | 826 (5.8%) | 45 (3.3%) | 871 (5.6%) |
| *Normal Weight* | 8,453 (59.2%) | 883 (65.2%) | 9,336 (59.7%) |
| *Overweight* | 3,263 (22.9%) | 294 (21.7%) | 3,557 (22.8%) |
| *Obese* | 1,736 (12.2%) | 132 (9.7%) | 1,868 (11.9%) |
| Smoking before pregnancy | 5,717 (42.0%) | 435 (33.7%) | 6,152 (41.3%) |
| Nicotine use before pregnancy | 1,070 (7.2%) | 108 (7.7%) | 1,178 (7.2%) |
| Alcohol before pregnancy | 4,037 (28.5%) | 418 (31.1%) | 4,455 (28.8%) |
| Illicit substance use before pregnancy | 228 (1.5%) | 13 (0.9%) | 241 (1.5%) |
| Folate use before pregnancy | 6,047 (40.5%) | 727 (52.1%) | 6,774 (41.5%) |
| Folate use during pregnancy | 12,060 (80.8%) | 1,244 (89.1%) | 13,304 (81.5%) |
| Life-time history of major depression in mothers | 9,157 (65.2%) | 1,115 (83.5%) | 10,272 (66.8%) |
| Obstetric comorbidity index |  |  |  |
| *0* | 9,884 (66.2%) | 867 (62.1%) | 10,751 (65.9%) |
| *1-2* | 3,534 (23.7%) | 374 (26.8%) | 3,908 (23.9%) |
| *3-5* | 1,428 (9.6%) | 150 (10.7%) | 1,578 (9.7%) |
| *>5* | 79 (0.5%) | 5 (0.4%) | 84 (0.5%) |
| Co-medication with psychotropics |  |  |  |
| *Opiods analgesics* | 300 (2.0%) | 27 (1.9%) | 327 (2.0%) |
| *Benzodiazedine derivates* | 218 (1.5%) | 34 (2.4%) | 252 (1.5%) |
| *Hypnotics and sedatives* | 167 (1.1%) | 33 (2.4%) | 200 (1.2%) |
| *Antipsychotics* | 89 (0.6%) | 12 (0.9%) | 101 (0.6%) |
| *Antiepileptics* | 96 (0.6%) | 10 (0.7%) | 106 (0.6%) |
| *No* | 14,055 (94.2%) | 1,280 (91.7%) | 15,335 (94.0%) |
| Paternal age categories |  |  |  |
| *<25 years* | 1,013 (6.8%) | 61 (4.4%) | 1,074 (6.6%) |
| *25-35 years* | 9,726 (65.6%) | 879 (63.5%) | 10,605 (65.4%) |
| *36-46 years* | 3,812 (25.7%) | 408 (29.5%) | 4,220 (26.0%) |
| *>46 years* | 268 (1.8%) | 37 (2.7%) | 305 (1.9%) |
| Life-time history of major depression in father | 2,196 (20.4%) | 229 (19.9%) | 2,425 (20.3%) |
| Child sex |  |  |  |
| *Female* | 7,144 (48.0%) | 730 (52.3%) | 7,874 (48.3%) |
| *Male* | 7,749 (52.0%) | 666 (47.7%) | 8,415 (51.7%) |
| Civil status |  |  |  |
| *Married* | 6,621 (44.6%) | 691 (50.0%) | 7,312 (45.1%) |
| *Co-habitant* | 7,270 (49.0%) | 621 (44.9%) | 7,891 (48.7%) |
| *Unmarried/Single* | 874 (5.9%) | 68 (4.9%) | 942 (5.8%) |
| *Divorced/Separated/Widow* | 65 (0.4%) | 2 (0.1%) | 67 (0.4%) |
| Mother income |  |  |  |
| *None* | 505 (3.5%) | 36 (2.6%) | 541 (3.4%) |
| *Low* | 4,965 (34.6%) | 437 (32.0%) | 5,402 (34.3%) |
| *Average* | 7,488 (52.1%) | 762 (55.8%) | 8,250 (52.5%) |
| *High* | 1,405 (9.8%) | 131 (9.6%) | 1,536 (9.8%) |
| Paternal income |  |  |  |
| *None* | 203 (1.5%) | 15 (1.1%) | 218 (1.4%) |
| *Low* | 1,899 (13.6%) | 151 (11.4%) | 2,050 (13.4%) |
| *Average* | 7,502 (53.7%) | 756 (56.8%) | 8,258 (54.0%) |
| *High* | 4,360 (31.2%) | 408 (30.7%) | 4,768 (31.2%) |
| Maternal education |  |  |  |
| *Secondary school* | 748 (5.3%) | 39 (3.0%) | 787 (5.1%) |
| *High school* | 5,689 (40.5%) | 400 (30.6%) | 6,089 (39.7%) |
| *University* | 7,604 (54.2%) | 867 (66.4%) | 8,471 (55.2%) |
| Paternal education |  |  |  |
| *Secondary school* | 863 (6.1%) | 59 (4.4%) | 922 (5.9%) |
| *High school* | 7,157 (50.2%) | 584 (43.6%) | 7,741 (49.7%) |
| *University* | 6,225 (43.7%) | 696 (52.0%) | 6,921 (44.4%) |
